# Estimation of genomic breeding values using the Horseshoe prior

**DOI:** 10.1186/1753-6561-8-S5-S6

**Published:** 2014-10-07

**Authors:** Ricardo Pong-Wong

**Affiliations:** 1 The Roslin Institute and the R(D)SVS, The University of Edinburgh, Easter Bush, Midlothian, EH25 9RG, Scotland, UK

## Abstract

**Background:**

A method for estimating genomic breeding values (GEBV) based on the Horseshoe prior was introduced and used on the analysis of the 16^th ^QTLMAS workshop dataset, which resembles three milk production traits. The method was compared with five commonly used methods: Bayes A, Bayes B, Bayes C, Bayesian Lasso and GLUP.

**Methods:**

The main difference between the methods is the prior distribution assumed during the estimation of the SNP effects. The distribution of the Bayesian Lasso is a Laplace distribution; for Bayes A is a Student-t; for Bayes B and Bayes C is a spike and slab prior combining a proportion of SNP without effect and a proportion with effect distributed as a Student-t or Gaussian for Bayes B and C, respectively; for GBLUP is similar to a ridge regression. The distribution for the Horseshoe prior behaves like log(1+1/β^2^) (up to a constant). It has an infinite spike at zero and heavy tail that decay by β^-2 ^(slower than the Laplace or the Student-t). The implementation of all methods (except GBLUP) was done using a MCMC approach, where the relevant parameters defining the prior distributions were jointly estimated from the data. The GBLUP was done using ASREML.

**Results:**

The accuracy for all methods ranged from 0.74 to 0.83, representing an improvement of 44% to 78% over the traditional BLUP evaluation. GEBV with the highest accuracy were obtained with Bayes A, Bayes B and the Horseshoe prior. The Horseshoe tended to select smaller number of SNP and assigning them larger effects, while strongly shrinking the remaining SNP to have an effect closer to zero.

**Conclusions:**

The Horseshoe prior showed a different shrinkage pattern than the other methods. While for this specific dataset, this has little impact on the accuracy of the GEBV, it may prove a good property to discriminate true effect from noise, and thereby, improve overall prediction under different scenarios.

## Background

Genomic selection can be described as the use of high dense genotyping for the evaluation of individuals to increase the accuracy of their estimated breeding values (GEBV) [1]. Several approaches and methods have been proposed and applied to the analysis of real data. Most of these methods use a regression-based approach where the genotype scores for all SNP are jointly fitted in the model (i.e. Ridge, Bayes A, Bayes B, Bayes C, Bayesian Lasso [1-3]). The SNP effects are calculated and thereafter the GEBV for each individual is calculated as the sum of all SNP effects, given their genotypes. These methods are defined by their choice of the prior distribution of the SNP effect, which induce shrinkage on the estimated effects to prevent over-parameterisation due to the large number of SNP fitted in the model. They are commonly implemented under a Gibbs sampling framework where the parameters defining the prior distribution in use can be jointly estimated in the analysis. The choice of the prior distribution affects the shrinkage pattern observed on the estimated SNP effects, which in turn, would impact on the variable selection properties of the method itself and their overall prediction performance.

In this study the Horseshoe distribution [4,5] is proposed as a prior for the SNP effect to estimate GEBV. The method was used to analyse the common dataset from the 2012 QTLMAS workshop, and the GEBV were compared with those estimated with the Bayes A, Bayes B, Bayes C, Bayesian Lasso and the GBLUP methods.

## Methods

### 1. Dataset

The dataset, provided by the 16^th ^QTLMAS workshop organisers, consisted of 3,000 individuals across three generations (G1-G3) phenotyped and genotyped for about 10,000 SNPs on five chromosomes of equal length. The phenotypes (Trait1, Trait2, and Trait3) resembled three milk production traits, given as individual yield deviations, and generated in order to mimic two yields and the corresponding content. Additionally, 1020 genotyped individuals from the fourth generation were used as the validation set to estimate GEBV.

### 2. Genomic evaluation

#### Linear model

The model for all methods is the following:

$$y=\mu +\overset{n}{\underset{i}{\sum }}z_{i}\beta_{i}+e$$

where, *n *is total number of SNP; **y **the vector of phenotypic observation; **z**_i _the vector of genotypes at SNP *i*; *β*_i _indicates the allelic substitution effect for SNP *i*.

The different methods are defined by the prior distribution of the SNP effects P(β) and they have been reported elsewhere (e.g. see [1-3,6]). Basically, the prior distribution for the Ridge regression is a normal distribution; for Bayesian Lasso is a Laplace distribution; for Bayes A is a scaled Student-t; for Bayes B is a '*spike-and-slab*' prior [7] where a proportion (π) of the SNP have a nonzero effect and distributed as a Student-t and the remaining (1-π) have no effect on the trait; for Bayes C the distribution is similar to Bayes B but the proportion of the SNPs with effect is assumed be normal distributed.

In order to facilitate the implementation of Lasso, Bayes A and Bayes B, the distribution of SNP effects is reformulated as a scale mixture of normal distributions [8]. The P(β) is obtained by expressing the effect of SNP *i *(i.e. P(β*_i_*)), as being distributed N(0, σ*_i_*^2^) and σ*_i_*^2 ^being a sample from a specific mixing distribution (P(σ *_i_*^2^)) to target a given P(β). Hence, the distribution P(σ*_i_*^2^) for Lasso is an exponential distribution defined by a scale/rate parameter; and for Bayes A and Bayes B is a scaled inverted χ^2^, defined by a scale and a shape parameter. In both cases, the conditional distribution for the scaling parameters σ*_i_*^2 ^is a known distribution making easy its implementation in a Gibbs sampling framework.

The Ridge regression method is commonly reformulated and known as GBLUP (e.g. see [9]) and easily implemented using a frequentist REML/BLUP framework.

#### The Horseshoe prior

The horseshoe prior was proposed and described by Carvalho *et al *[4,5] as having good properties to discriminate between true effect and noise. Assuming this prior, the distribution of the SNP effect P(β) behaves like α log(1+1/β^2^) (up to a constant). It has an infinite spike at zero and heavy tail that decay by β^-2 ^(slower than the Laplace or the Student-t). This characteristic means that (compared with GBLUP, Bayesian Lasso, Bayes A and Bayes B) the Horseshoe prior would apply greater shrinkage to smaller effects but much less to larger ones. Such behaviour may prove to be a useful property to discriminate between true effects and noise (and thereby, improve the predictions).

Similar to previous distribution, the horseshoe prior is implemented using a scale mixture of normal, where the mixing scale distribution is a half Cauchy prior and applied on σ*_i _*(not σ*_i_*^2^). Therefore:

• P(β) α log(1+1/β^2^)

• P(β*_i_*)= N(0, σ*_i_*^2^)

• P(σ*_i_*) = C^+^(0, τ)

• P(τ)= C^+^(0,ζ),

where C^+^(0,a) is the standard half Cauchy distribution on the positive side with scale parameter a. For the prior distribution to estimate ζ Carvalho *et al *[4,5] suggested the Jeffrey's prior (i.e p(a) α 1/a), whereas Scott [10] fix ζ to be =1. Here, to make the results from the Horseshoe prior more comparable with the other methods, a bounded flat prior was used on τ^2 ^instead of the Cauchy distribution on τ (this is because τ^2 ^would be equivalent to the scale parameter associated to P(σ*_i_*) for the Bayesian Lasso, Bayes A and Bayes B methods, and they were assumed to have the a bounded flat prior).

#### Implementation

The GBLUP was implemented using ASREML [11], in a two-step approach where the variance components are first estimated from the data and later used to calculate the GEBV.

The models Bayes A, Bayes B, Bayes C, Bayesian Lasso and the horseshoe prior were implemented under a Bayesian framework using Gibbs sampling. The conditional distributions required for the Bayes A, Bayes B, Bayes C and Lasso can be found elsewhere (e.g.[3,12]). For the horseshoe prior, the conditional distribution for σ*_i_* does not have close form so the sampling of these parameters was done using a slice sampling approach [10,13] similar to the one proposed by Scott [10].

The parameters defining the prior distribution of the SNP effect were also estimated in the analysis (i.e. the scale/rate parameter for all methods, the shape parameter for Bayes A and Bayes B, the proportion of SNPs with effect for Bayes B and Bayes C). Bounded flat priors were used for all these parameters.

For each analysis, a MCMC chain was run and the first 50,000 cycles were discarded as burn-in period. Following this, 50,000 realisations were collected, each separated by 50 cycles between consecutive realisations (i.e. length of chain = 2,550,000 cycles). The posterior mean was used as the estimate for each parameter of interest. Genetic variance for all genomic selection methods (except GBLUP) was calculated from the GEBV using an approximation based on the infinitesimal model theory. Assuming that GEBV for all training individuals have the same prediction error variance (PEV), the genetic variance is calculated as the sum of the variance of the GEBV plus the mean PEV for all training individuals (see [2]). The environmental variance was calculated using the realisations from the MCMC chain. Both variance estimates were later used to estimate the heritability of the trait in question.

## Results and discussion

Heritability estimates were very similar across the methods, with the Bayesian Lasso yielding slightly larger estimates than the others and GBLUP having the lowest ones (Table 1). All methods slightly underestimated the true heritabilities used for simulating the data (i.e. 0.36, 0.35 and 0.52 for the three traits, respectively).

**Table 1:** Estimated heritabilities, slope of regression of TBV on GEBV and accuracies of GEBV for the genomic selection and standard BLUP methods.

		Method						
		
		Horseshoe	BayesA	BayesB	BayesC	B Lasso	GBLUP	BLUP
Trait 1	h^2^	0.295	0.311	0.310	0.302	0.333	0.308	0.382
	slope	1.061*	1.061*	1.064*	1.072*	1.110*	1.164*	1.000
	*r*^a^	0.791	0.793	0.794	0.789	0.766	0.738	0.459
	Impr^b^	72.4%	72.9%	73.1%	72.0%	67.0%	60.9%	

Trait 2	h^2^	0. 303	0. 313	0. 312	0. 300	0. 341	0.318	0.387
	slope	1.018	1.030	1.030	1.021	1.095*	1.162*	1.114
	*r*^a^	0.825	0.834	0.833	0.820	0.809	0.771	0.534
	Impr^b^	54.6%	56.3%	56.1%	53.6%	51.6%	44.4%	

Trait 3	h^2^	0.460	0.471	0.471	0.452	0.527	0.470	0.486
	slope	1.022	1.029	1.029	1.012	1.033	1.083*	1.010
	*r*^a^	0.824	0.828	0.828	0.817	0.791	0.760	0.464
	Impr^b^	77.7%	78.7%	78.7%	76.2%	70.7%	63.9%	

a: Pearson correlation between true and estimated breeding value for training population. b: percentage of improvement in the correlation over standard BLUP. *: Slope of regression significantly different from 1 (*p*<0.05)

**Figure 1:**
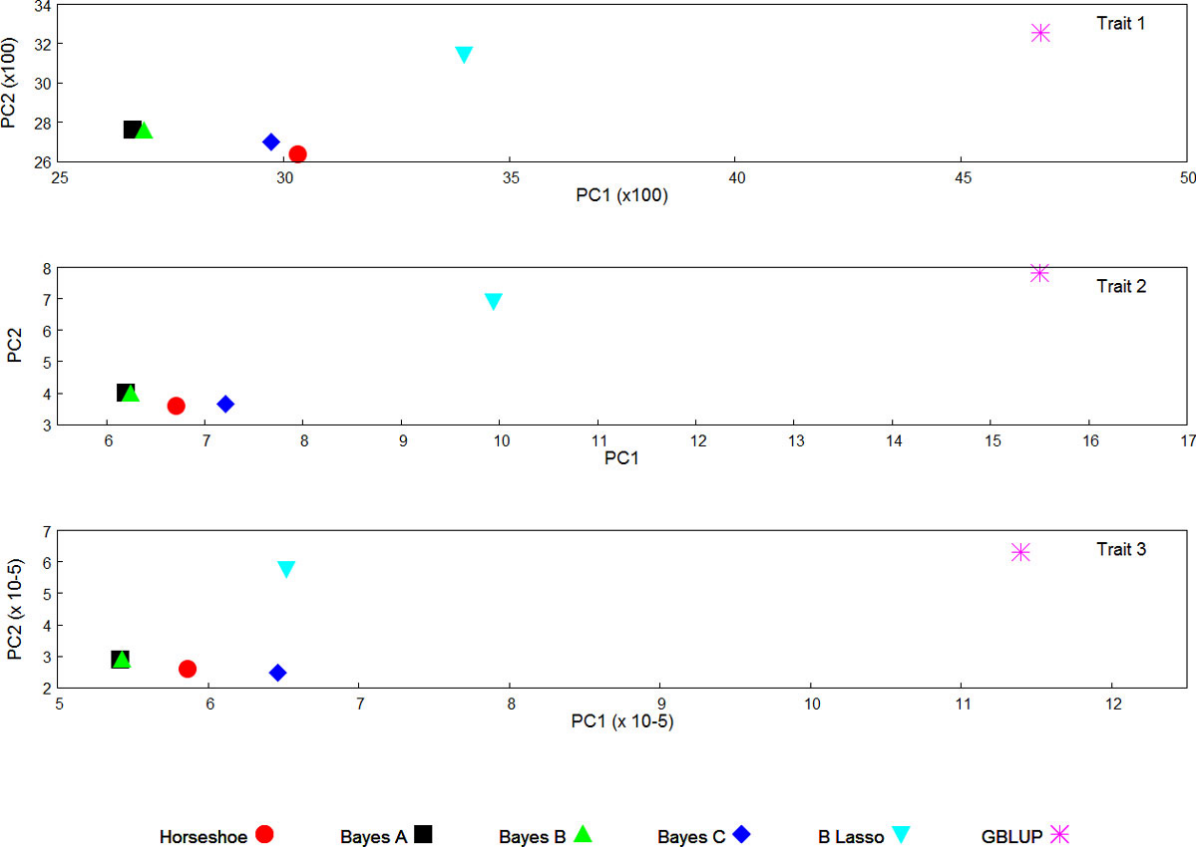
**Principal component analysis on GEBV for the three traits, obtained with the different genomic selection methods**. The values of the two largest principal components were rebased so the position corresponding to true breeding values is centred at the origin of the graph (hence the accuracy of the methods relates to their closeness to the origin of the graph).

Overall, the GEBV were consistent across the different methods, with correlations ranging from 0.84 to 1 (see Additional File 1). The most correlated methods were the Bayes A, Bayes B and Horseshoe methods, where the correlations among any pair of them was always greater than 0.98. The relationship between the different methods is better illustrated with the results from a principal component analysis carried out on the GEBVs (Figure 1). For all three traits, the two largest principal components clustered together Bayes A and Bayes B; the Horseshoe prior tended to be located between Bayes C and the Bayes A/Bayes B cluster; and the Bayesian Lasso and GBLUP were the most distant methods. Similar pattern between the GEBV of these methods when the parameters of the prior distribution are jointly estimated with the GEBV has been reported before [2].

The observed accuracy of the GEBV (measured as their correlation with the true breeding values, TBV) ranged between 0.74 and 0.83, across all methods and traits, representing an improvement between 44% and 78 % compared with the accuracies obtained with the traditional BLUP evaluation (Table 1). Across the three traits, the highest accuracies were observed with the Horseshoe, Bayes A and Bayes B methods, followed by Bayes C, Bayesian Lasso and GBLUP. The reduction in accuracy with Bayes C was marginal, but the reduction observed with the Bayesian Lasso and GBLUP was more noticeable. In order to quantify the bias associated with the different methods, the TBV were regressed on their GEBV, and the results shown in Table 1. Some of the regression slopes (especially for GBLUP and Bayesian Lasso) were significantly different from the optimal value of 1. But this deviation was very small in most trait-method combinations, which suggests that any potential biases on the GEBV would be very small for the evaluation methods tested here. The slopes for the Horseshoe prior were the closest to the optimal value of 1, so the method with the least bias when calculating the GEBV.

**Figure 2:**
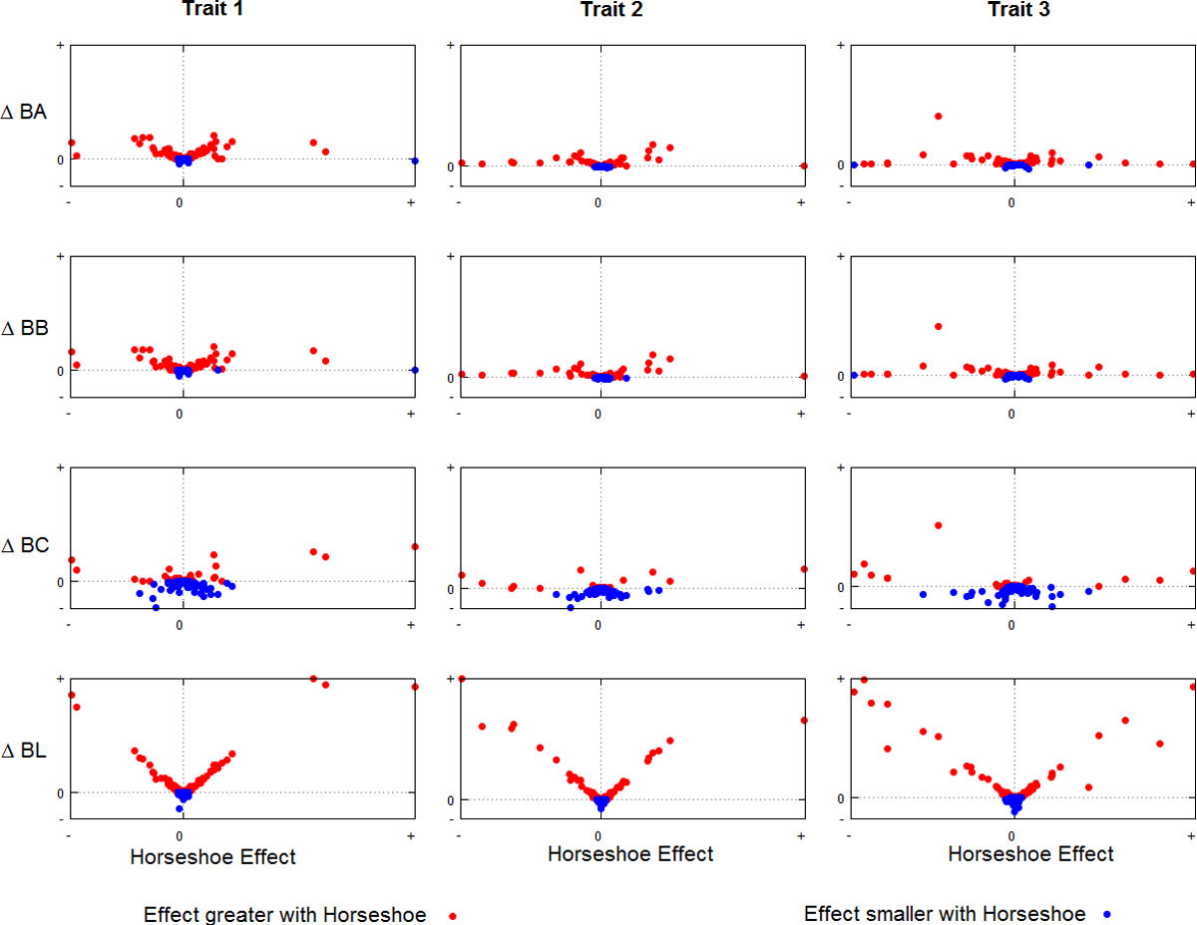
**Scatter plots between the Horseshoe estimated SNP effects and effects estimated with other methods (expressed as the difference of their absolute effects and the Horseshoe estimates)**. Positive differences mean that estimates from the Horseshoe have greater magnitude than the alternative method.

Although the overall GEBV obtained with the Horseshoe prior, Bayes A and Bayes B were very similar, the differences on the individual estimated SNP effects were large, suggesting differences in the shrinkage properties of the methods. Figure 2 shows the scatter plots between the Horseshoe's estimated SNP effects and the effects estimated with the other methods (expressed as the difference of their absolute effects and the Horseshoe estimates, so a positive deviation means that the Horseshoe's estimated SNP effect has greater magnitude). Across the three traits, the general trend shows that when the SNP has a large effect, the magnitude of the Horseshoe estimates were larger than those estimated from the other methods (i.e. differences were positive), but when the SNP effects were close to zero, the Horseshoe estimates were further shrinked to zero. This characteristic of the Horseshoe prior to apply greater shrinkage to smaller effects but much less to larger ones, would be a useful property of the method to discriminate between true effects and noise. For this particular dataset, the differences on shrinkage pattern between methods have little impact on the overall accuracy of the GEBV, but under other situations the characteristic of the Horseshoe prior may prove to be of great value to improve overall predictions. For instance, scenarios where the actual mutations affecting the traits are genotyped (or are in strong LD with genotyped SNP) may benefit more from methods with stronger discrimination between potential true effects and noise. Hence, the performance of the Horseshoe prior may improve with denser SNP genotyping or whole sequence data.

## Conclusions

The accuracy of the GEBV from the different genomic selection methods ranged between 0.74 and 0.83, representing an improvement of 44% to 78% over the traditional BLUP evaluation. The highest GEBV accuracies were observed with the Horseshoe, Bayes A and Bayes B methods. Although they yielded very similar GEBV, the route for estimating them was different. The Horseshoe prior tends to assign greater effect to fewer SNPs, while strongly shrinking smaller effect. This property may prove beneficial in situations using much denser SNP genotyping or whole sequence data.

## Competing interests

The author declares that they have no competing interests.

## Supplementary Material

Additional file S 1 **Pearson correlation between GEBV obtained with the different methods of genomic selection**. Click here for file

## Declarations

This work and its publication were funded by the BBSRC Institute Strategic Grant BB/J004235.

This article has been published as part of *BMC Proceedings *Volume 8 Supplement 5, 2014: Proceedings of the 16th European Workshop on QTL Mapping and Marker Assisted Selection (QTL-MAS). The full contents of the supplement are available online at http://www.biomedcentral.com/bmcproc/supplements/8/S5
