# Supplementary material for: Estimation of genomic breeding values using the Horseshoe prior
Source: BMC Proc. 2014 Oct 7;8(Suppl 5):S6. doi: 10.1186/1753-6561-8-S5-S6 (PMC4195408; doi:10.1186/1753-6561-8-S5-S6)
Supplement: Additional file S 1 — Pearson correlation between GEBV obtained with the different methods of genomic selection. [file 1753-6561-8-S5-S6-S1.docx]

Table S1. Pearson correlation between GEBV obtained with the different methods of genomic selection

|  | Horseshoe | BayesA | BayesB | BayesC | B Lasso | GBLUP |
| --- | --- | --- | --- | --- | --- | --- |
| Trait 1 | | | | | | |
| Horseshoe | 1.000 |  |  |  |  |  |
| BayesA | 0.992 | 1.000 |  |  |  |  |
| BayesB | 0.994 | 1.000 | 1.000 |  |  |  |
| BayesC | 0.993 | 0.985 | 0.987 | 1.000 |  |  |
| B Lasso | 0.909 | 0.944 | 0.943 | 0.920 | 1.000 |  |
| GBLUP | 0.859 | 0.895 | 0.894 | 0.876 | 0.982 | 1.000 |
| Trait 2 | | | | | | |
| Horseshoe | 1.000 |  |  |  |  |  |
| BayesA | 0.997 | 1.000 |  |  |  |  |
| BayesB | 0.998 | 1.000 | 1.000 |  |  |  |
| BayesC | 0.996 | 0.991 | 0.993 | 1.000 |  |  |
| B Lasso | 0.903 | 0.926 | 0.925 | 0.901 | 1.000 |  |
| GBLUP | 0.843 | 0.866 | 0.866 | 0.847 | 0.977 | 1.000 |
| Trait 3 | | | | | | |
| Horseshoe | 1.000 |  |  |  |  |  |
| BayesA | 0.998 | 1.000 |  |  |  |  |
| BayesB | 0.998 | 1.000 | 1.000 |  |  |  |
| BayesC | 0.994 | 0.987 | 0.989 | 1.000 |  |  |
| B Lasso | 0.893 | 0.911 | 0.911 | 0.880 | 1.000 |  |
| GBLUP | 0.848 | 0.868 | 0.867 | 0.834 | 0.980 | 1.000 |
